# Prevalence and Factors Associated with Current Cigarette Smoking among Ethiopian University Students: A Systematic Review and Meta-Analysis

**DOI:** 10.1155/2020/9483164

**Published:** 2020-04-21

**Authors:** Yonas Deressa Guracho, Getachew Setotaw Addis, Sileshi Mulatu Tafere, Kidanu Hurisa, Berhanu Boru Bifftu, Martha H. Goedert, Yared Mulu Gelaw

**Affiliations:** ^1^Bahir Dar University, College of Medicine and Health Science, Department of Psychiatry, Bahir Dar, Ethiopia; ^2^Bahir Dar University, College of Medicine and Health Science, Department of Health Informatics, Bahir Dar, Ethiopia; ^3^Bahir Dar University, College of Medicine and Health Science, Department of Nursing, Bahir Dar, Ethiopia; ^4^ICAP at Columbia University, Bahir Dar, Ethiopia; ^5^University of Gondar College of Medicine and Health Science, School of Nursing, Gondar, Ethiopia; ^6^Bahir Dar University, University of Nebraska College of Public Health, Omaha, Nebraska, USA; ^7^Bahir Dar University, College of Medicine and Health Science, Department of Public Health, Bahir Dar, Ethiopia

## Abstract

**Background:**

Although tobacco use is highest in high-income countries, most tobacco-related deaths occur in low- and middle-income countries with the highest number of deaths recorded in East Africa. The aim of this systematic review and meta-analysis is to determine the pooled prevalence and associated factors of current cigarette smoking among Ethiopian university students.

**Methods:**

The authors searched databases from PubMed, PsycINFO, Google Scholar, EMBASE, and Web of Sciences. The publications included in the analysis were inclusive, the literature was searched from January 2011 to December 2018. The JBI-MAStARI critical appraisal tool was applied to 13 publications identified by the database search. I^2^ statistics were used to indicate heterogeneity. Publication bias was evaluated using the visual funnel plot. A *p* value < 0.1 was considered as indicative of statistically significant publication bias. A random effect meta-analysis model was computed to estimate the pooled prevalence of cigarette smoking, and the variables associated with cigarette smoking were examined.

**Results:**

The meta-analysis of 13 studies showed that the pooled prevalence of current cigarette smoking was found to be 12.55% (95% CI: 10.39–14.72; I^2^ = 94.0%) with no publication bias according to Egger's test (*p* = 0.007) for cigarette smoking by 2.05 (95% CI: 1.52–2.75). Factors associated with cigarette smoking were peer influence 2.79 (95% CI: 1.62–4.82; I^2^ = 35.7), khat chewing (95% CI: 2.81–15.26; I^2^ = 82.5), and alcohol use 11.16 (95% CI: 7.46–16.71).

**Conclusion:**

Our findings indicate a high prevalence of cigarette smoking among Ethiopian university students as compared to the general population. Gender, peer influence, khat chewing, and current alcohol use were significantly associated with cigarette smoking. The authors recommend promoting antismoking campaigns, emphasizing health hazard public service announcements about cigarettes, and integrating health education on smoking in youth-friendly services, especially targeting higher educational institutions.

## 1. Background

Tobacco smoke is a toxic mix of more than 7,000 chemicals, and many of these chemicals are stored or detoxified in body's tissues where they cause damage. Over time, the damage can lead to disease [1]. Tobacco-related respiratory diseases are distressing; they affect the quality of life of all ages and are responsible for over 7 million deaths globally [2]. Currently, tobacco smoking impacts approximately 20% of total adult mortality worldwide [3]. Although tobacco use is highest in high-income countries [4], most tobacco-smoking related deaths occur in low-income and middle-income countries [5].

Approximately six percent of global health expenditures are attributed to smoking, with the additional burden experienced by developing countries with 40% of health care expenditures related to smoking [6]. Cigarette smoking by youth and young adults has immediate adverse health consequences including addiction and chronic disease acceleration across the entire life course [7]. The burden of tobacco-smoking related deaths in Africa has increased rapidly from 150,000 reported deaths in 1990 to over 215,000 in 2016. The majority of tobacco-related deaths are recorded in East Africa [8].

In Ethiopia, 3.7% (2.5 million) of adults are currently smoking (6.2% among men and 1.2% among women) [9]. Nearly one-third of adult Ethiopians are exposed to secondhand smoke at work [10]. More than half of the noncommunicable disease (NCD) burden could be avoided through effective health promotion and disease prevention programs that tackle risk factors with low-cost and highly efficacious initiatives to curb tobacco use [11].

The WHO targeted a 30% relative reduction in tobacco use among persons aged 15 years and older by 2025 in the Sustainable Development Goals (SDGs). To date, there have been less than half of these goals met with a 14% reduction in prevalence rates globally. The global push to achieve a significant decrease in tobacco use is now critical if the goal of reaching a 30% reduction by 2025 is attained [12]. In Ethiopia, the projected prevalence of smoking by 2025 will be four percent [13]. Accordingly, the Ethiopian Federal Ministry of Health had set a national target of 15% reduction of tobacco use among persons aged 15 and above by 2020. In respect to current trends, Ethiopia will not achieve WHO's target to reduce tobacco use by 30% by 2025 [14]. Cigarette smoking by youth and young adults has immediate and long-term adverse health consequences including death. Despite the critical impact of tobacco use, there is a gap in data concerning university student smoking habits and the population to target with risk reduction. It is estimated that Ethiopian university students' cigarette smoking ranges from of 9.00%–29.5%. The groups most likely to smoke are characterized by male sex, by khat chewing, by alcohol use, and by peer pressure influence. These preliminary-associated demographic and behavioral factors have been observed in university settings by health care professionals. However, there are no Ethiopian university-based data that quantify or describe the populations and associated characteristics and behaviors of students who use tobacco. A systematic review and meta-analysis aimed to estimate the overall prevalence of current cigarette smoking and its associated factors among Ethiopian university students is critically needed. The data can inform policy makers, educators, and health care workers on the ways to help reduce the prevalence of university student tobacco use and, in the end, to improve the health of Ethiopians.

### 1.1. Systematic Review Questions

What is the best available evidence on the prevalence and associated factors of cigarette smoking among Ethiopian university students?

## 2. Objective

The objective of this study is to determine the prevalence of cigarette smoking and its associated factors.

## 3. Methods

### 3.1. Reporting and Search Strategy

The researchers conducted a systematic review in accordance with the preferred reporting items for systematic review and meta-analysis [15]. Potentially relevant studies were identified through a literature search of electronic databases such as PubMed, PsycINFO, and Web of science. Unpublished studies have been retrieved from the gray literature through Google and Google Scholar.

### 3.2. Selection of Studies

All studies retrieved through the search strategy were imported to EndNote X7 (Thomson Reuters, New York, USA). Duplicated studies were removed from EndNote library. The title and abstract of the remaining articles were assessed independently by five reviewers (YDG, GSA, SMT, KH, and BBB). Disagreements were resolved by taking the mean score of the four reviewers after discussing rationale about differences in judgment and repeating the review procedure.

### 3.3. Definition of Concepts

In this study, current cigarette smoking was defined as having smoked at least once in the last 30 days. The prevalence of cigarette smoking was determined by dividing the total number currently smoking by the overall number of participants in the studies.

### 3.4. Eligibility Criteria

- The searches were limited by date of publication from January 2011 to December 2018
- Qualified research must have estimated the prevalence of current smoking in the sample and must be carried out among Ethiopian university students. Studies other than English language and studies reporting prevalence of cigarette smoking among general populations, clinical patients, and prisoners were excluded from review.

### 3.5. Outcome Measure

The systematic review with meta-analysis had two main outcomes. The first outcome was to determine the pooled prevalence of current cigarette smoking. The second outcome of the study was to identify factors associated with cigarette smoking. The prevalence of cigarette smoking was calculated by dividing the number of students engaged in cigarette smoking to the total number of students who have been included in the study (total sample size) multiplied by 100. For the associated factors, the reported odds ratio, 95% confidence interval, and *p* value were used.

### 3.6. Study Design

Observational studies (cross-sectional and cohort/longitudinal) were included. Studies that focused on case reports and conference abstracts were excluded.

### 3.7. Data Extraction

Data were extracted from the eligible studies using a preconceived and piloted data abstraction form by four independent authors (YDG, GSA, KH, and BBB). The extracted data include the name of the first author, region, university where the studies were carried out, year of publication, study design, sample size, number of smokers (prevalence of cigarette smoking), and associated factors.

### 3.8. Quality Assessment

Five authors (YDG, GSA, SMT, BBB, MHG, and YMG) appraised the quality of each included study using a Joanna Briggs Institute's Meta-Analysis of Statistics Assessment and Review Instrument (JBI-MAStARI) which is a critical appraisal tool for prevalence and analytical cross-sectional studies. The JBI-MAStARI has eight components needed to qualify: (1) clearly defined inclusion in the sample, (2) study subjects and the setting described in detail, (3) exposure measured in a valid and reliable way, (4) objective, standard criteria used for measurement of the condition, (5) identified confounding factors, (6) strategies to deal with confounding factors stated, (7) outcomes measured in a valid and reliable way, and (8) appropriate statistical analysis used.

Studies which fulfill all eight components were included as cases [16, 17] (Table 1).

### 3.9. Data Synthesis and Statistical Analysis

The extracted data from the eligible studies were entered into a Microsoft Excel Database and were converted to event/count, prevalence/proportion, standard error, lower boundary of prevalence (LBPV), upper boundary of prevalence (UBPV), odds ratios (ORs), Lan of odds ratio (lnOR), and standard error odds ratio (SEOR) and imported into STATA version 14 for analysis. Meta-analysis was performed by “Metaprop” command using random-effects models with the DerSimonian and Laird method-based transformed values and their variance. The Freeman–Tukey variant of the arcsine square root transformation of proportions was fitted to avoid variance instability when handling proportions close to one. The magnitude of heterogeneity between studies was measured by the index of the heterogeneity (I^2^ statistics) test. I^2^ values of 25%, 50%, and 75% was used as low, medium, and high heterogeneity, respectively. Subgroup analysis was performed based on region where the university was located to determine any existing difference in current cigarette smoking prevalence between regions in Ethiopia. Publication bias was evaluated using the visual funnel plot. A *p* value of <0.1 was considered as indicative of statistically significant publication bias. We also performed sensitivity analysis to identify heterogeneity of studies [18–20]. For the analysis of associated factors, the reported odds ratio, 95% confidence interval, and *p* value were used. Meta-analysis of the associated factors was performed for those studies with at least two studies reported the same associated factors.

## 4. Results

### 4.1. Description

**Figure 1:**
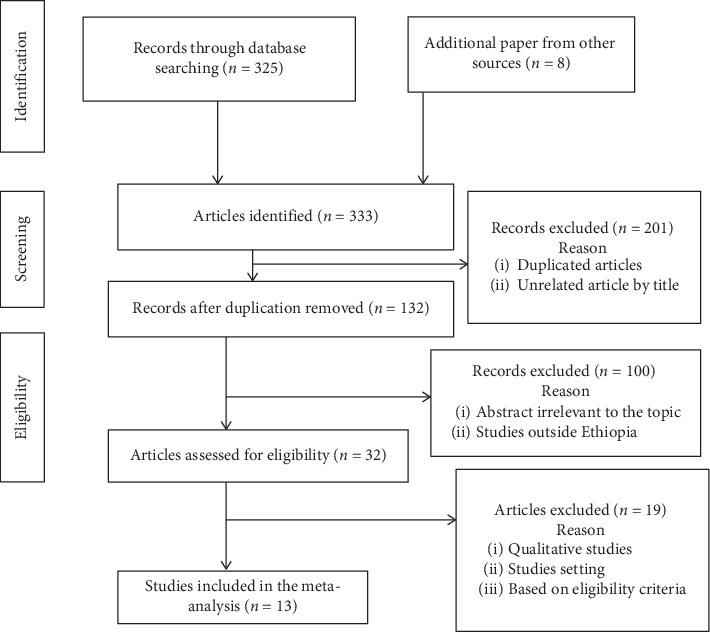
Flow diagram of the studies included in the meta-analysis.

All records identified through database searching were located from July 1, 2019, to September 10, 2019. Out of 332 studies, sixty were considered for analysis. The researchers excluded 47 studies using PRISMA 2009 Flow Diagram [21] (Figure 1).

### 4.2. Study Characteristics

In this study, a total of 7,861 study participants were included from 13 studies. These studies were carried out from January 2011 to December 2018 from 10 different universities in seven regions of the country: Addis Ababa = 1, Amhara = 3, Dire Dawa = 1, Oromia = 1, Somali = 2, SNNPR = 3, and Tigray = 1 [22–34]. All of the studies included were cross-sectional with a sample size ranging from 271 to 1022 (Table 1). Based on the JBI-MAStARI, all the included studies had no methodological defect.

### 4.3. Prevalence of Current Cigarette Smoking

**Figure 2:**
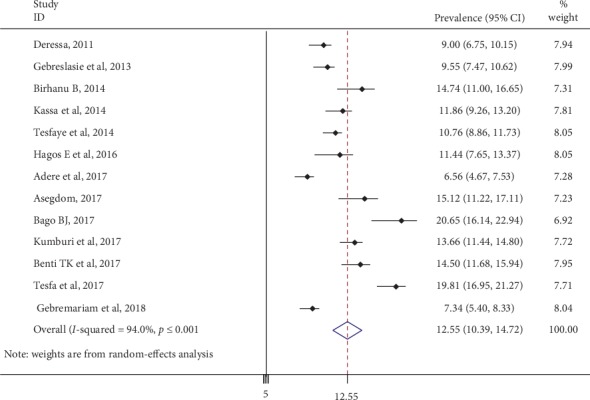
Forest plot of the pooled prevalence of current cigarette smoking among Ethiopian university students.

**Figure 3:**
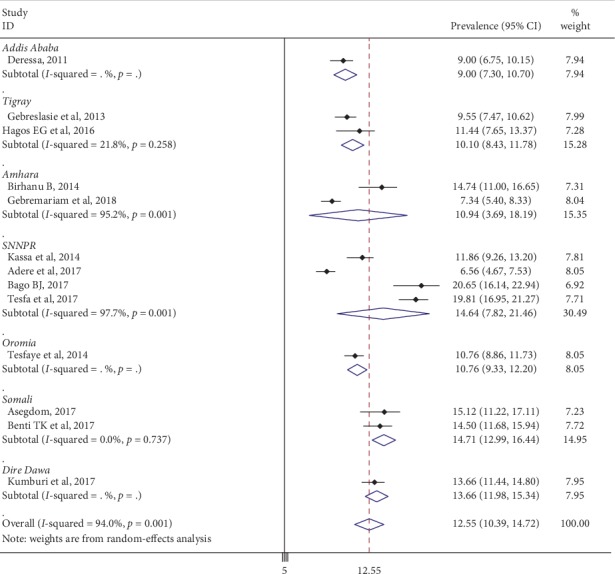
The forest plot showing the subgroup analysis of the prevalence of current cigarette smoking among Ethiopian university students.

The pooled prevalence of current cigarette smoking was found to be 12.55% (95% CI: 10.39, 14.72; I^2^ = 94%, *p* ≤ 0.001) among Ethiopian university students (Figure 2). To see symmetry of publications, the funnel plot was applied, and the visual inspection of funnel plot showed there was no publication bias (Figure 3), and Egger's test showed there is no change in the trim and fill analysis (*p* < 0.007).

**Figure 4:**
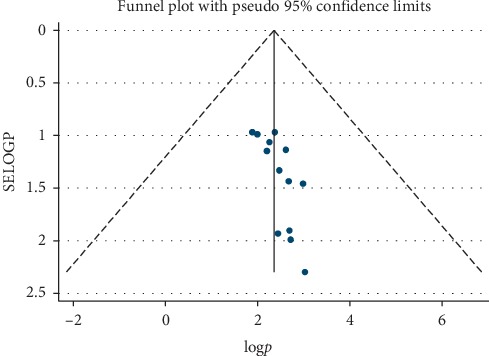
Funnel plot presenting the visual inspection of publication bias for systematic review and meta-analysis of cigarette smoking among Ethiopian university students.

**Figure 5:**
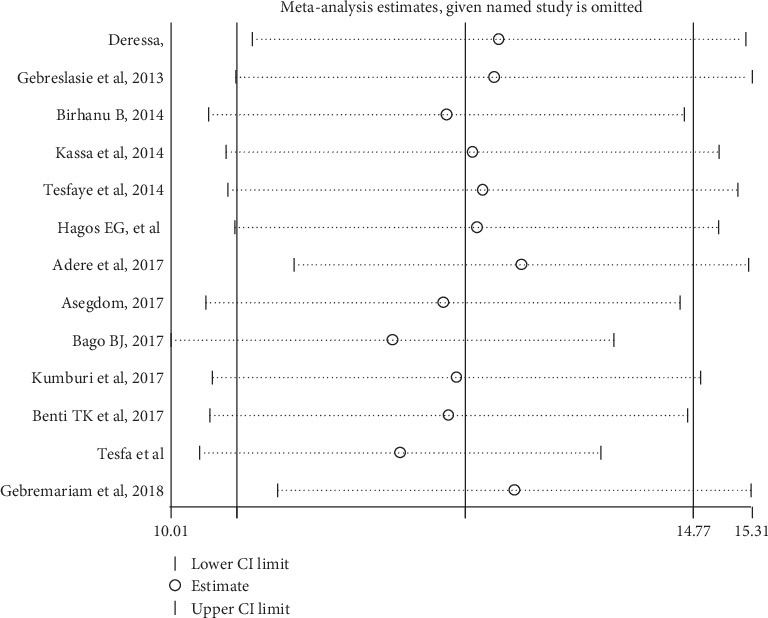
Sensitivity analysis for studies included in a systematic review and meta-analysis of cigarette smoking among Ethiopian university students.

Subgroup analysis based on the region of university was done. The result revealed that the prevalence of current cigarette smoking was 9% in Addis Ababa, 10.10% in Tigray Region, 10.94% in Amhara Region, 14.64% in SNNPR Region, 10.76% Oromia Region, 14.7% in Somali Region, and 13.66% in Dire Dawa (Figure 4). However, the heterogeneity among regions of universities remained high (Figure 5).

### 4.4. Factors Associated with Cigarette Smoking among Ethiopian University Students

In this study, being male, khat chewing, peer pressure, and alcohol use were associated with current cigarette smoking among Ethiopian university students.

The pooled effect of three studies showed that male students were two times more likely to experience cigarette smoking (2.05 (95% CI: 1.52–2.75; I^2^ = 0.0%)) [22, 23, 31] as compared to female students.

The results of three studies revealed that those students who had friends with history of cigarette smoking were 2.79 times more likely to smoke cigarettes as compared to those who did not have a peer group of friends smoking (2.79 (95% CI: 1.62–4.82; I^2^ = 35.7)) [22, 23, 31].

**Figure 6:**
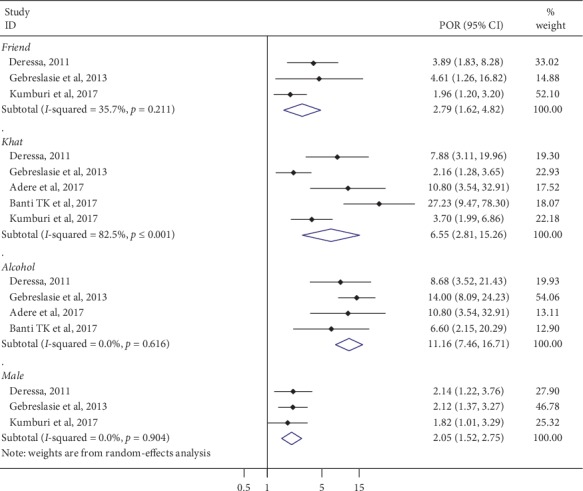
A systematic review and meta-analysis of factors associated (being male, peer influence, khat chewing, and alcohol use) with cigarette smoking among Ethiopian university students.

From the pooled effect size of four studies, those students who had history of khat chewing were 6.55 times more likely to smoke cigarettes as compared to nonkhat chewers (6.55 (95% CI: 2.81–15.26; I^2^ = 82.5)) [22, 23, 31, 32]. The finding of this study also showed that those students who had history of alcohol use were 11.2 times more likely to smoke cigarettes (11.16 (95% CI: 7.46–16.71; I^2^ = 0.0%)) [22, 23, 28, 32] (Figure 6).

## 5. Discussion

To the best of our knowledge, this systematic and meta-analysis is the first to estimate the pooled prevalence and associated factors of cigarette smoking among Ethiopian university students. The purpose of this systematic review and meta-analysis was to estimate the pooled prevalence and factors associated with cigarette smoking among Ethiopian university students. The results of this finding showed that the pooled prevalence of current cigarette smoking was found to be 12.55% (95% CI: 10.39, 14.72; I^2^ = 94.0%). There was regional variation in pooled prevalence between universities with cigarette smoking ranging from 9% in Addis Ababa to 14.64% in SNNPR Region with substantial heterogeneity between studies. The highest prevalence of cigarette smoking was reported in the Somali Region universities with 14.71% of the students smoking (95% CI: 12.99, 16.44; I^2^ = 00). This might be explained by the regional culture of students attending their regional universities. For example, tobacco and khat are more affordable and khat has deep-rooted social and cultural meaning for some communities in the Somali Region compared to the Tigray Region.

This finding is similar to studies carried out globally with smoking prevalence reported at the University of East Africa as 9.02% [35]. Meta-analyses from Mainland Chinese and from the Kingdom of Saudi Arabia reported, respectively, university student smoking prevalence at 10.83% [36] and 17% [37]. Those articles were specific to medical and health science college students. King Faisal University in Saudi Arabia reported student smoking prevalence at 28.1% [38] Other universities have targeted interventions, considering regional variations [39]. The possible justification for these variations could be due to the population's sociocultural values and norms as well as religious beliefs [40, 41]. Addiction to substances in general starts a cascade of poor academic achievement, compromised health, added expenses related to cigarette use, and possible long-term addiction. The ill effects of smoking if addressed early with effective educational and campus support activities can reduce cigarette smoking and create a culture that does not condone use in campus facilities and community spaces [33]. Special attention should be given to universities when implementing the policies and interventions to reduce cigarette smoking.

Regarding the associated factors, the pooled adjusted odds ratios showed being male students were two times 2.05 (95% CI: 1.52–2.75; I^2^ = 0.0%) more likely to have smoked cigarettes as compared to female students. This is in line with other similar meta-analysis globally [37, 38, 42–44].

Those students who have peers with a history of smoking were almost three times more likely to have smoked cigarettes 2.79 (95% CI: 1.62–4.82; I^2^ = 35.7) as compared to those students who did not have peers who were smoking. This is consistent with other studies carried out in East Africa [35, 45]. During adolescence and early adult years, individuals have a higher predisposition to imitate and exercise what they observe in their peer group.

Tobacco use is embedded within the culture of khat chewing [46]. The habit of tobacco use among khat users is substantial [47]. In this study, current khat and alcohol use were significantly associated with smoking cigarettes compared to students' counterparts. These results lend further support to the peer influence on habits related to cigarette smoking. The results support specific interventions that address specific factors and target high-risk groups for both primary and secondary prevention of cigarette smoking.

### 5.1. Limitations of This Study

Only English articles or reports were considered. This meta-analysis represented only studies reported from universities; therefore, we did not evaluate studies from college and technical or vocational training centers. All studies included in this review were cross-sectional in nature; as a result, the outcome variable might be affected by other confounding variables.

## 6. Conclusions

Our finding indicated high prevalence of current cigarette smoking among Ethiopian university students as compared to general population. Being male, having peers who smoke, and actively using khat and/or alcohol are factors significantly associated with current cigarette smoking. The authors suggest the universities' higher officials need to raise awareness through public service announcements and curriculum-based education the facts associated with the adverse effects from cigarette smoking. It is possible to reduce smoking among university students while addressing cofactors such as khat chewing and alcohol use particularly for those students having peer with history of substance use. In addition, policies on campus concerning smoking restrictions in community spaces and university facilities may help reduce both onset of smoking and reduction or discontinuation of smoking for students who smoke. In the long run, these interventions and policies emphasize health promotion and disease prevention related to smoking. Action to promote a smoke-free student population can impact the future health of Ethiopians, many who will be the country's future leaders, scholars, and professionals.

## Figures and Tables

**Table 1:** Characteristics of 13 studies reporting prevalence of current cigarette smoking among Ethiopian university students included in systematic review and meta-analysis of 2019.

Region of university	Name of university	Author	Year of publication	Design	Data collection method	Sample size	Prevalence (%)
Addis Ababa	Addis Ababa University	Deressa and Azazh	2011	Cross sectional	Self-administered	622	9.00

Tigray	Axum University	Gebreslassie et al.	2013	Cross sectional	Self-administered	764	9.50
	Sheba University	Hagos et al.	2016	Cross sectional	Self-administered	271	11.40

	Debre Berhan University	Gebremariam et al.	2018	Cross sectional	Self-administered	695	7.40

Amhara	Woldia University	Adere et al.	2017	Cross sectional	Self-administered	655	6.40

	Debre Berhan University	Birhanu B.	2014	Cross sectional	Self-administered	346	14.70

SNNPR	Hawassa University	Bago BJ.	2017	Cross sectional	Self-administered	310	20.60
	Hawassa University	Kassa et al.	2017	Cross sectional	Self-administered	590	11.90
	Wolaita University	Mekonen et al.	2017	Cross sectional	Self-administered	747	19.80

Dire Dawa	Dire Dawa University	Kumburi et al.	2017	Cross sectional	Self-administered	915	13.70

Oromia	Haramaya University	Tesfaye et al.	2014	Cross sectional	Self-administered	1022	10.80

Somali	Jijiga University	Asgedom T.	2017	Cross sectional	Self-administered	324	15.20
	Jijiga University	Banti TK et al.	2017	Cross sectional	Self-administered	600	14.50

**Table 2:** Sensitivity analysis of the prevalence of current cigarette smoking among Ethiopian university students.

Study omitted	Prevalence (95% confidence interval)
Deressa, 2011	13.00 (10.75–15.25)
Gebreslasie et al., 2013	12.95 (10.59–15.30)
Birhanu B, 2014	12.52 (10.35–14.69)
Kassa et al., 2014	12.76 (10.51–15.00)
Tesfaye et al., 2014	12.85 (10.52–15.17)
Hagos EG et al., 2016	12.79 (10.59–15.00)
Adere et al., 2017	13.20 (11.13–15.27)
Asegdom, 2017	12.49 (10.34–14.65)
Bago BJ., 2017	12.03 (10.013–14.05)
Kumburi et al., 2017	12.61 (10.38–14.83)
Benti TK et al., 2017	12.54 (10.36–14.72)
Tesfa et al., 2017	12.09 (10.27–13.93)
Gebremariam et al., 2018	13.14 (10.97–15.29)
Combined	**12.69 (10.61–14.77)**

## Abbreviations

CI: Confidence interval
MBASE: Excerpta medica database
I^2^: Index of heterogeneity
MEDLINE: Medical literature analysis and retrieval system online
MeSH: Medical subject heading
OR: Odds ratio
PRISMA-P: Preferred reporting items for systematic review and meta-analysis protocols.

## References

[1] Department of Health and Human Services U.S. (2010). *A Report of the Surgeon General: How Tobacco Smoke Causes Disease: What it Means to You*.

[2] WHO Don’t let tobacco take your breath away. https://www.who.int/docs/default-source/world-no-tobacco-day/wntb-2019-brochure.pdf?sfvrsn=deac371c_22.

[3] Rentería E., Jha P., Forman D., Soerjomataram I. (2016). The impact of cigarette smoking on life expectancy between 1980 and 2010: a global perspective. *Tobacco Control*.

[4] (2018). *World Health Statistics 2018: Monitoring Health for the SDGs, Sustainable Development Goals*.

[5] (2017). GBD 2016 Risk Factors Collaborators. Global, regional, and national comparative risk assessment of 84 behavioural, environmental and occupational, and metabolic risks or clusters of risks, 1990–2016: a systematic analysis for the Global Burden of Disease Study 2016. *Lancet*.

[6] Goodchild M., Nargis N., Tursan d’Espaignet E. (2018). Global economic cost of smoking-attributable diseases. *Tobacco Control*.

[7] Department of Health and Human Services U.S. (2012). *Preventing Tobacco Use Among Youth and Young Adults: A Report of the Surgeon General*.

[8] Magitta NwF: N. F. (2018). Epidemiology of tobacco use and dependence in Sub-Saharan Africa: a systematic review. *International Journal of Pulmonology & Clinical Research*.

[9] Executive---Summary_GATS_Ethiopia_Oct-20_2017

[10] Abiye Y. (2017). Ethiopia’s first tobacco survey reveals alarming trend. https://www.thereporterethiopia.com/article/ethiopias-first-tobacco-survey-reveals-alarming-trend-bahir-dar-bans-khat.

[11] The World Bank: Human Development Network (2011). The growing danger of non-communicable diseases. *Acting Now to Reverse Course*.

[12] WHO (2018). *Global Report on Trends in Prevalence of Tobacco Smoking 2000–2025*.

[13] WHO (2015). *Global Report on Trends in Prevalence of Tobacco Smoking*.

[14] Ethiopia Tobacco Control Strategic Plan 2010-2012 E.C(2017/18-2019/20): Addis Abeba, 2017

[15] Moher D., Shamseer L., Clarke M (2015). Preferred reporting items for systematic review and meta-analysis protocols (PRISMA-P) 2015 statement. *Systematic Reviews*.

[16] Munn Z., Moola S., Lisy K., Riitano D., Tufanaru C. (2015). Methodological guidance for systematic reviews of observational epidemiological studies reporting prevalence and cumulative incidence data. *International Journal of Evidence-Based Healthcare*.

[17] Moola S. M. Z., Tufanaru C., Aromataris E, Aromataris E., Munn Z. (2017). Chapter 7: systematic reviews of etiology and risk. *Joanna Briggs Institute Reviewer’s Manual*.

[18] Egger M., Smith G. D., Schneider M., Minder C. (1997). Bias in meta-analysis detected by a simple, graphical test. *BMJ*.

[19] Duval S., Tweedie R. (2000). Trim and fill: a simple funnel-plot-based method of testing and adjusting for publication bias in meta-analysis. *Biometrics*.

[20] Liu J. L. (2011). The role of the funnel plot in detecting publication and related biases in meta-analysis. *Evidence-Based Dentistry*.

[21] Moher D. L. A., Tetzlaff J., Altman D. G. (2009). The PRISMA group preferred reporting items for systematic reviews and meta-analyses: the PRISMA statement. *PLoS Med*.

[22] Deressa W., Azazh A. (2011). Substance use and its predictors among undergraduate medical students of Addis Ababa University in Ethiopia. *BMC Public Health*.

[23] Gebreslassie M., Feleke A., Melese T. (2013). Psychoactive substances use and associated factors among Axum University students, Axum Town, North Ethiopia. *BMC Public Health*.

[24] Birhanu B. (2014). *The Relationship between Stress, Coping Behaviour and Substance Abuse Among*.

[25] Kassa A., Taddesse F., Yilma A. (2014). prevalence-and-factors-determining-psychoactive-substance-pas-use-among-hawassa-university-hu-undergraduate-students-hawassa-Ethiopia. *BMC Public Health*.

[26] Tesfaye G., Derese A., Hambisa M. T. (2014). Substance use and associated factors among university students in Ethiopia: a cross-sectional study. *Journal of Addiction*.

[27] Hagos E. G., Asfeha G. G., Berihu B. A. (2016). Prevalence of substance abuse among regular degree health science students in Sheba University College in Mekelle Town, Tigray - Ethiopia. *Journal of Neurosciences in Rural Practice*.

[28] Adere A., Yimer N. B., Kumsa H., Liben M. L. (2017). Determinants of psychoactive substances use among Woldia University students in Northeastern Ethiopia. *BMC Research Notes*.

[29] Asgedomt T. T.: Substance Abuse Among Undergraduate Students at a University in Ethiopia. 2017

[30] Bago B. J. (2016). Prevalence of cigarette smoking and its associated risk factors among students of hawassa university, college of medicine and health sciences. *Journal of Addiction Research & Therapy*.

[31] Tesso Kumburi G. (2017). Psycho-active substances use and determining factors among regular undergraduate students of dire-dawa university, eastern Ethiopia. *Science Journal of Public Health*.

[32] Banti T. K., Mengesh D. S., Mamade G. F. (2017). Prevalence of cigarette smoking and factors associated with it among undergraduate students of jigjiga university. *International Journal of Psychological and Brain Sciences*.

[33] Tesfa Mekonen W. F., Tefera Chane Mekonnen S. B. W. (2017). Substance use as a strong predictor of poor academic achievement among university students. *Hindawi: Psychiatry Journal*.

[34] Gebremariam T. B., Mruts K. B., Neway T. K. (2018). Substance use and associated factors among Debre Berhan University students, Central Ethiopia. *Subst Abuse Treat Prev Policy*.

[35] Tezera N., Endalamaw A. (2019). Current cigarette smoking and its predictors among school-going adolescents in East Africa: a systematic review and meta-analysis. *International Journal of Pediatrics*.

[36] Niu L., Liu Y., Luo D., Xiao S. (2018). Current smoking behavior among medical students in Mainland China: a systematic review and meta-analysis. *Asia Pacific Journal of Public Health*.

[37] Alotaibi S. A., Alsuliman M. A., Durgampudi P. K. (2019). Smoking tobacco prevalence among college students in the Kingdom of Saudi Arabia: systematic review and meta-analysis. *Tobacco Induced Diseases*.

[38] Al-Mohamed H. I., Amin T. T. (2010). Pattern and prevalence of smoking among students at king faisal university, Al hassa, Saudi Arabia. *Eastern Mediterranean Health Journal*.

[39] Ayalew M., Tafere M., Asmare Y. (2018). Prevalence, trends, and consequences of substance use among university students: implication for intervention. *International Quarterly of Community Health Education*.

[40] Prabhu A., Obi K. O., Rubenstein J. H. (2013). Systematic review with meta-analysis: race-specific effects of alcohol and tobacco on the risk of oesophageal squamous cell carcinoma. *Alimentary Pharmacology & Therapeutics*.

[41] Koenig H. G. (2001). Religion and medicine II: religion, mental health, and related behaviors. *The International Journal of Psychiatry in Medicine*.

[42] Haghdoost A. A., Moosazadeh M. (2013). The prevalence of cigarette smoking among students of Iran’s universities: a systematic review and meta-analysis. *Journal of Research in Medical Sciences*.

[43] Brathwaite R., Addo J., Smeeth L., Lock K. (2015). A systematic review of tobacco smoking prevalence and description of tobacco control strategies in sub-saharan african countries; 2007 to 2014. *PLoS One*.

[44] Townsend L., Flisher A. J., Gilreath T., King G. (2006). A systematic literature review of tobacco use among adults 15 years and older in Sub-Saharan Africa. *Drug and Alcohol Dependence*.

[45] Bandason T., Rusakaniko S. (2010). Prevalence and associated factors of smoking among secondary school students in Harare Zimbabwe. *Tobacco Induced Diseases*.

[46] Nichter M. (2003). Smoking: what does culture have to do with it?. *Addiction*.

[47] Kassim S., Jawad M., Croucher R., Akl E. A. (2015). The epidemiology of tobacco use among khat users_ a systematic review. *BioMed Research International*.

